# Research trends, developments, and future perspectives in brand attitude: A bibliometric analysis utilizing the Scopus database (1944–2021)

**DOI:** 10.1016/j.heliyon.2022.e12765

**Published:** 2022-12-30

**Authors:** Alharthi Rami Hashem E, Nor Zafir Md Salleh, Mazilah Abdullah, Adnan Ali, Faisal Faisal, Roshazlizawati Mohd Nor

**Affiliations:** a Department of Financial and Administrative Sciences, Ranyah University College, Taif University, P. O. Box 11099, Taif, 21944, Saudi Arabia; b Universiti Teknologi Malaysia, Skudai, 81310, Johor, Malaysia; c Department of Management Sciences, Shaheed Benazir Bhutto University, Sheringal, Dir Upper, 18050, Khyber Pakhtunkhwa, Pakistan; d Institute of Business Studies and Leadership, Abdul Wali Khan University, Mardan, Khyber Pakhtunkhwa, Pakistan

**Keywords:** Brand attitude, Implicit brand attitude, Explicit brand attitude, Bibliometric analysis, Scientometrics analysis

## Abstract

Brand attitude has been the primary concern for corporation sustainability for a long. Studies have focused on various attributes concerning brand attitude comprising of many research articles. This study focuses on bibliometric analysis of brand attitude examining 1497 documents published for the years 1944–2021. The purpose of this study is to examine historic research trends, developments, and future conduits based on co-authorship and co-occurrence analysis using VOSviewer. The study highlights the most prolific articles containing the most productive authors and their affiliations, keywords, and influential journals, along with future research trends. The result shows that the number of publications follows a very slow trail until 2008, mildly until 2012, following which the cumulative growth in publication increased manifold. Further, results show that researchers from the United States, South Korea, and Taiwan extended many efforts to the global knowledge of literature leading other countries. The emerging hotspot keywords analysis suggests that brand avoidance, subtle, brand betrayal, consumer ethnocentrism, environmental sustainability and policy, brand activism, brand authenticity, consumer brand engagement, and brand competence are the key areas that attract researchers' attention policymakers as future research boulevards. In the case of consumers' brand attitudes, there is a scarcity of studies that investigated the consumers' implicit and explicit brand attitudes.

## 1 Introduction

Brand attitude has been one of the most widely examined constructs in consumer behavior [1,2]. Brand attitude refers to a customer's reaction to and preference for a brand, and it is essential for forecasting customer responses to marketing activities (Howard, 1994). Customers' brand attitudes are driven by their familiarity with and confidence in a brand; the more significant their familiarity and trust, the greater their expected purchasing intention (Ramesh, 2019). If the brand performs well, customers will be satisfied and they will create a positive attitude and eventually develop loyalty with a consistently good performance by the brand [3,4]. Brand attitude is an important element as it is being used to envisage customers buying preferences Brand attitude reveals consumers' preferences, serving as a helpful indicator of their propensity to purchase products and brand loyalty [5]. Brand attitude is the overall assessment of the brand, requiring cognition and fitting, which results in behavioral intention [6].

Despite its importance in the business and consumer world, the research area is rich in empirical studies while it faces deficiency towards comprehensive literature review or bibliometric analysis. In other words, a comprehensive performance analysis of scientific actors and bibliometric knowledge mapping has not been conducted. The obvious reason as depicted in Fig. 2 illustrates that, the cumulative growth in publication is just a decade old. With the advent in technology and digital advertising campaigns such as social media in addition to print and electronic media, diverted the researcher's inclination more towards empirical studies only, thus leaving a gap in the literature. Moreover, a time span and number of empirical, case studies, theoretical and conceptual studies are required to conduct any systematic literature review or bibliometric analysis which was not possible earlier with fewer number of publications. Furthermore, the technological advancement concerning brand attitude such as a direct outreach to the consumers, accessing their feedback, addressing and adhering to consumers demand [7,8], led to the spike of publications which changed the dynamics of the conventional literature. Such development in the empirical literature put forward a research gap to encourage and conduct comprehensive literature review or bibliometric analysis in order to identify the emerging trends and future hotspots concerning brand attitude. Thus, holding this opportunity, our study tried to bridges this gap to provide fruitful insights into the past literature, explore current trends, and identify future aspects regarding brand attitude. Specifically, this article focuses on this research gap based on no literature review and no bibliometric or scientometric review in this field. It is pertinent to mention that the current article is only focused on bibliometric analysis and systematic literature review is left for future research. Usually, researchers in every academic field review previous relevant and quality studies, put forth the crux matter, and highlight what has been done. However, such studies are limited in the number of documents reviewed and suffer from subjectivity biases [[9], [10], [11]]. Moreover, due to the bulk number of publications and time limitations, it is humanly impossible to review all the literature. Therefore, the solution is laid in adopting the Scientometrics and Bibliometric analysis [[12], [13], [14]].

More importantly, bibliometrics help researchers distinguish in required fields, the core academic institutions, core journals, core articles, envisage development trends by evaluating the current state of the literature [15,16]. The authors, therefore, searched for any bibliometric, scientometric, or science mapping study using Google Scholar, Web of Science, and the Scopus database but could not succeed and returned with no bibliometric study in this area. Hence, despite its theoretical, practical importance, and the tremendous growth in literature, the theme lacks a bibliometric approach. Therefore, this bibliometric study shall facilitate mapping the research area in an easy and understandable pattern for many users such as beginner researchers, various organizations, several funders, sponsors, practitioners, and other academicians and social scientists.

Therefore, keeping in view the instant literature and the importance of the phenomenon of interest, this study adopted scientometric and bibliometric analysis to explain the historical trends and prospects considering the central theme “Brand Attitude” using the leading research database i.e., the Scopus Database. The Scopus database is considered the largest citation and abstract database covering a wide range of subjects compared to the Web of Science database [17,18]. To the authors' best knowledge, this study has utilized all the keywords used by various authors, arranging them in a query search string with proper use of field codes and Boolean operators; however, there may be certain limitations [19,20]. Hence, this study shall serve as the first bibliometric and scientometric study in the area as mentioned earlier of interest and could serve as the foundation on which future research can be built. To address the knowledge gaps, specifically, this study shall answer the questions regarding, a) what are the historical trend and developments in the avenue of brand attitude? b) who are the most prolific authors and academic institutions and their geographical placement on google map? c) what could be the way forward for researchers, policymakers, and organizations. In addition, this article also provides provision of online real data access to readers to ensure, foresee and explore more than what is described here due to space constraints.

To answer these questions and achieve the study objectives the current study adopted bibliometric analysis approach comprising of both qualitative and quantitative performance analysis in addition to science mapping-intellectual and conceptual structures [21–23]. According to Ref. [23]; this allow us to quantify and visualize the thematic evolution of the research field. Additionally, productivity of scientific actors such as number of documents, journals, authors, and countries are analyzed for quantitative measures and for qualitative measures-quality based indices such as citations received and other indices such as h-index is measured [23,24]. Consequently, such study can elaborate on area's evolution, illustrating current trends, developments and identify future aspects [22], regarding brand attitude. Thus, the study is relevant and presents novelty of the bibliometric analysis tool in terms of its contribution in the brand attitude research. Moreover, specifically the contributions of the current study are derived-in light of the research questions, from the descriptive analysis of the documents, authors, journals and prolific institutions which are further augmented by the emerging trends, hotspot keywords and niche areas identified and put forth for future research.

## 2 Literature review

This section narrates the relevant and critical literature concerning brand attitude. Brand attitude is the consumer perception of a good or service as determined by market research. Any product or service's success on the market is greatly influenced by its brand. Researchers in marketing literature have been attempting to understand how consumers evaluate brands and respond to various branding strategies [25]. Consumers' general likes and dislikes can be thought of as attitudes toward a brand. Companies need to stand out, and a company's brand offers the chance to showcase its brand reputation. Brand attitude is a consumer's overall assessment of that brand and is an important concept for both businesses and consumers [26] and therefore, it has an influence on purchase intention [27]. A customer's brand attitude also tends to reveal his or her predisposition to recommending the brand to others. The significant overall value offered to the customer will enhance the brand's and business' market share and profitability. Consequently, Brand attitudes are therefore an important measure in determining possible buying behavior among current and potential customers [28].

The theory of reason action (TRA) describes the relationship between attitude and behavior (Ajzen, 1991) and posits that humans are rational. According to TRA, attitude is a psychological tendency to develop an opinion on a particular item to a certain degree, and behavior is a function of an individual's attitude [27]. Brand attitude is critical since it is used to forecast consumer purchasing choices (Chaudhuri, 1999). Businesses can identify their most loyal customers and thus conduct additional research into their brand preferences. It identifies consumers' preferences and dislikes; hence, it serves as a helpful indicator of consumers' purchasing intention and brand loyalty (Ramesh, 2019) and (Foroudi, 2018). Market share and profitability of the brand and company will increase as a result of the high total value offered to the customer. As a result, brand attitude has become more significant in today's market [29]. The development of the brand attitude also communicates the overall perception of what the brand means to current and potential customers, in addition to satisfying consumer needs.

Brand attitude is an important concept for both businesses and consumers [26]. Brand attitude emerges as a result of brand exposure, which is also known as brand knowledge. Consumers shape a brand's functional and symbolic qualities through physical product and service experience or marketing content. Customers that have a positive brand attitude toward a brand are more likely to pay a premium price for it [30] and a consumer-brand relationship can be maintained by accumulating positive brand attitudes [31] research has also established brand attitude as an important driver of brand equity. Furthermore, if a customer has a favorable opinion toward a brand, as well as a negative attitude toward competitor brands, these will influence their decision to buy the brand [32]. As a result, in order to add value to their offerings, businesses must build positive brand attitude. The next section discusses the methodology along with tools and techniques used to conduct this bibliometric analysis.

## 3 Materials and methods

### 3.1 Software and tools used in the analysis

The current study engaged the Scopus database for the analysis. This study employed broadly two techniques, the ‘Preferred Reporting Items for Systematic Reviews and Meta-Analyses’ (PRISMA) for a systematic review of the articles included in the analysis and the Bibliometric Analysis for creating knowledge maps using VOSviewer software through co-citation and co-authorship analysis. In addition, this study also uses MS Excel 365, Google Maps, Windows Text Document (Notepad), and Endnote X9 software. Finally, this study uses the Pastebin and Bitly online tools to create online links for data and knowledge maps availability.

### 3.2 Data exploration and plan

Bibliometric analysis broadly comprises four stages, i.e., data acquisition, data pre-processing, statistical calculation, and application analysis. Before conducting any bibliometric analysis, the keywords are regarded as the foundation on which the analysis is based. For this purpose, the authors' studies, more than 100 articles both recently-earlier published and with a good number of citations, were obtained from the WOS and the Scopus Database to pen down important author keywords considered central theme as “Brand Attitude.” Afterward, these keywords are utilized in different combinations based on trial and error between August 2, 2021 until August 07, 2021 and finalized the following search query string. The authors saved the final search string and list of the documents for future needs.

TITLE-ABS-KEY (“Brand* Attitude*" OR “Implicit brand* attitude*" OR “explicit brand* attitude*" OR “consumer* brand* attitude*" OR “product attitude” OR “product* attitude*" OR “Implicit product* attitude*" OR “explicit product* attitude*") AND (LIMIT-TO (DOCTYPE, “ar”)) AND (LIMIT-TO (LANGUAGE, “English”)) AND (LIMIT-TO (SRCTYPE, “j")).

This query string initially resulted in 1721 documents, and after limiting to journal articles and English language, resulted in 1537 documents, following which the number increased by five more documents dated August 06, 2021, ending at 1727 documents and 1542 documents, respectively. This showed that it is temporal trend analysis, and over time, the number might increase due to more publishing from the authors. Hence our study is limited to the mentioned final date, i.e., August 07, 2021, and shows the first publication in the year 1944 indexed in the Scopus Database. Further, this study is limited to journal articles deemed the most prestigious academic publications [33]. Hence the first stage of data acquisition is accomplished.

**Figure 1:**
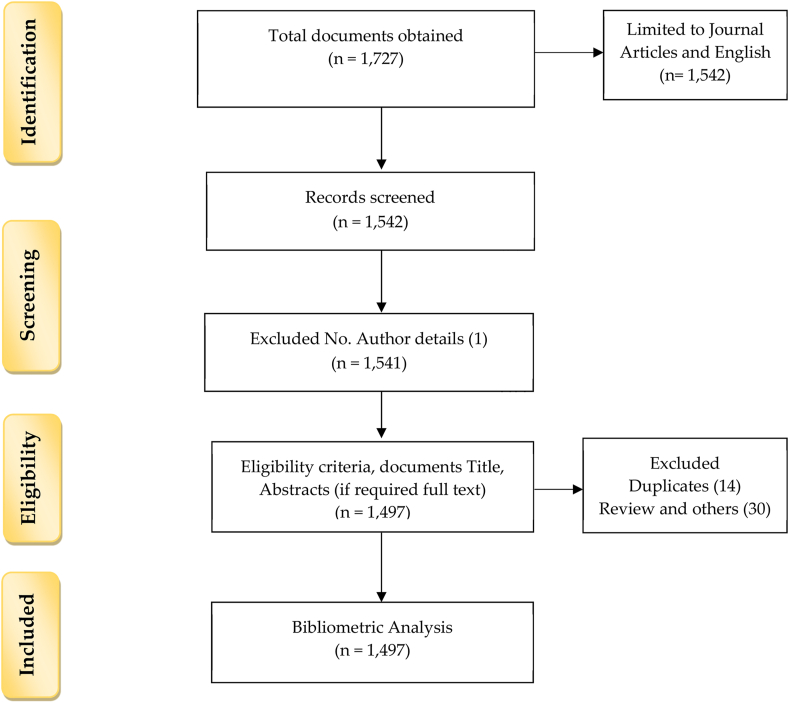
Flow diagram of PRISMA methodology and bibliometric analysis.

The next stage is data pre-processing which is obtained through PRISMA methodology as shown in Fig. 1 (please note that the authors only followed the steps in scrutiny and do not conduct systematic literature review as per PRISMA methodology) [34] and by following the study of [35]. This study identified documents 1727 initially, and finally 1542 for further screening. In the screening phase, one article is removed based on the absence of no authors details. In the eligibility phase, 1541 documents are screened using the Scopus database, MS Excel 365, and Endnote X9 for duplicates, potential review articles, and any other irrelevant articles by going through their titles, abstract, and if required, the full article, which resulted in total 34 documents. These articles were removed from the final dataset using their Electronic Identification (EID), a unique feature of the Scopus database. They finally reached 1497 documents for further data analysis. Hence the second stage of data pre-processing is completed.

**Figure 2:**
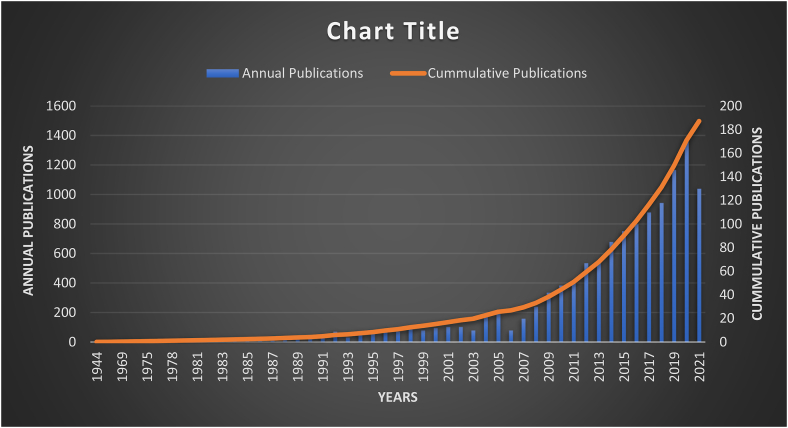
Annual and cumulative publications from 1944 to 2021 (Scopus Database).

The next stage involves statistical calculation comprised of overtime publication growth, developments, quantitative and descriptive analysis valuable in the bibliometric analysis [36]. This stage is based on using a combination of Scopus database, MS Excel 365, Endnote X9, and Google Map. These calculations illustrate the outcome in the form of illustrations of most prolific authors, their most cited articles, the most productive academic institutions, and their countries, the single country publications, and drawing their position on Google map for a global holistic view considering brand attitude. Hence the third stage of statistical calculation is completed.

The next stage involves application analysis. This stage involves the MS Excel 365, Windows Text Document (Notepad), Endnote X9, VOSviewer, Pastebin, and Bitly for creating knowledge maps, thesaurus files, and online links for results and data validation. This stage involves the bibliometrics analysis by employing the co-authorship and co-occurrence analysis using the countries and author keywords as a unit of analysis. This stage involves the incorporation of the final dataset of 1497 articles finalized via the steps mentioned by PRISMA flow diagram and exported to VOSviewer, which focuses on the holistic and timely developments in the phenomenon of interest illustrating the form of bibliometric knowledge maps [33,37]. The knowledge maps show the link between authors and their countries considering their collaborations and the co-occurrence of the most prominent keywords. Bibliometrics studies, besides other analyses, usually focus on the two types, i.e., co-authorship and co-occurrence analysis [38,39]. The former shows the link strength between two affiliated countries sharing the number of documents published, and the total link strength shows the total strength of a given country to other countries. The latter illustrates the link strength between author keywords portraying the keywords occurring together in the publications; for further details, look at [37].

Further, following on [40], the countries are clustered into seven regions for the current study, which is; South Asia, Africa, East Asia, and the Pacific, Europe and Central Asia, Latin America and the Caribbean, and the Middle East and North Africa, and North America. Furthermore, in the analysis of co-occurrence of author keywords, 3380 keywords from 1497 articles are obtained by VOSviewer. These keywords are analyzed for synonymic words or different variations demonstrating the same phenomenon before importing it into VOSviewer. The authors established the minimum occurrence of the keywords to 2 to provide a clear knowledge map resulting in 656 keywords. Finally, this stage also illustrates a table highlighting future research hotspots signaling potential unexplored areas for further exploration and collaboration. Hence the final stage of application analysis is completed.

## 4 Results and discussion

This part of the study answers the research questions mentioned earlier, considering the central theme of brand attitude. This section has only considered articles to develop a quality insight and produce excellent output suggesting valuable future recommendations.

### 4.1 Research growth trend considering brand attitude

This statistical analysis section illustrates the historical trend of quality articles published in the Scopus database considering the central theme of “Brand Attitude.” The results mentioned in Fig. 2 illustrates that the first article indexed in the Scopus database dates back to 1944. Since then, until recently, a total of 1497 articles has been produced and indexed in the Scopus database comprising 76 years by some 2986 authors in total. The results show very slow growth in the number of articles produced until 2008, which shows a more increasing trend in 2012 and onward. Moreover, articles in this area are published in many languages such as English, Spanish etc.; however, we limited our analysis to the English language only for brevity and understanding purposes. This suggests that the journals should publish the English version of the titles and abstracts to be understood by authors across the globe.

Moreover, publications in brand attitude are connected to multiple subject areas describing it as multidisciplinary. The subject area analysis depicts that the primary focus of the documents considering brand attitude is the Business, Management, and Accounting subject area with 1163 (47.5%) publications. Similarly, it is followed by social sciences (380–15.5%), psychology (208–8.5%), Economics, Econometrics and Finance (187–7.6%) etc. Besides these, the results also show publications in some interesting subject areas such as Engineering (42–1.7%), Medicine (39–1.6%), Energy (37–1.5%), signifying that the future of brand attitude is also linked to these areas and future research can be sought in this connection.

### 4.2 Prolific journals and articles

The results show that different publishers publish prolific journals considering the central theme of brand attitude. These include Taylor & Francis (04), Wiley-Blackwell (02), and others Elsevier, Emerald, Springer Nature, Oxford University Press own one journal. Table 1 [2,[41], [42], [43], [44], [45], [46], [47], [48], [49]], is listed based on the number of publications and illustrates the top journals, their most cited article, and other metrics. The top journal is the Journal of Business Research, having an impact factor of 7.550, with 92 publications (6.15%) and 3512 citations which Elsevier owns.

**Table 1:** Top 10-most prolific journals regarding brand attitude.

#	Journals with aIF 2020	No. of Articles Published	No. of Articles Published %	Citations	Cite Score 2020	Highly Cited Article	Citations	Publisher
1	Journal of Business Research (7.550)	92	6.15%	3512	9.2	[41]	399	Elsevier
2	Journal of Advertising (5.522)	68	4.54%	5662	11.3	[42]	548	Taylor & Francis
3	International Journal of Advertising (4.620)	58	3.87%	1723	6.2	[43]	313	Taylor & Francis
4	Journal of Product and Brand Management (4.335)	52	3.47%	574	4.7	[44]	102	Emerald
5	Psychology and Marketing (2.939)	50	3.34%	89	4.5	[45]	206	Wiley-Blackwell
6	Journal of Marketing Communications (Scopus)	45	3.01%	1095	4.6	[46]	244	Taylor & Francis
7	Journal of Brand Management (3.500)	36	2.40%	713	4.8	[2]	172	Springer Nature
8	Journal of Consumer Psychology (3.330)	34	2.27%	4077	5.8	[47]	1158	Wiley-Blackwell
9	Journal of Promotion Management (Scopus)	31	2.07%	297	2.7	[48]	34	Taylor & Francis
10	Journal of Consumer Research (7.000)	26	1.74%	2755	11.8	[49]	380	Oxford University Press

a Impact Factor.

Table 1 further illustrates that the second prolific journal is the Journal of Advertising (5.522), having an impact factor of 5.522, with 68 publications (4.54%) and 5662 citations owned by Taylor & Francis. The list is followed by the International Journal of Advertising (4.620), Journal of Product and Brand Management (4.335), Psychology and Marketing (2.939). Further, the Journal of Marketing Communications is not an impact factor journal indexed in the Scopus database. However, the commonality between the journals is that all are high-impact journals which shows the importance of the subject area under study. Besides the impact factor, the metric CiteScore is also considered as an alternative to the impact factor provided by Clarivate Analytics [50], which is used to measure journal impact based on citation data from the Scopus database [51]. The results of CiteScore (“calculates the average number of citations received in a calendar year by all items published in that journal in the preceding three years”) shows that Journal of Consumer Research (7.000), with CiteScore 11.8 at serial 10 is the top journal receiving high citations from the articles published in the journals indexed in Scopus database. The list is followed by the Journal of Advertising (5.522) with CiteScore 11.3 at serial two and the Journal of Business Research (7.550) with CiteScore 9.2 at serial 1. Based on citations received, the Journal of Advertising (5.522) with 5662 citations is the top journal followed by Journal of Consumer Psychology (3.330) with 4077, Journal of Business Research (7.550) with 3512, and Journal of Consumer Research (7.000) with 2755 citations.

### 4.3 Prolific authors

Table 2 demonstrates the ten prolific authors contemplating brand attitude, which are affiliated to 05 countries Belgium 03 authors, United States (02), Australia (02), Netherlands (02), Taiwan (01). Table 2 is listed based on the h-index calculated by the Scopus database for articles concerning brand attitude. It is essential to mention that the authors publish in many aligned subject areas whereas their total number of documents, citations, and h-index could be more than the one presented in Table 2; however, the current study is only focused on brand attitude and hence considering those articles and citations which are the result of our query search string. Based on this, Table 2 shows that the author, De Pelsmacker, P. is top of the list with 19 publications and h-index 10, having the first article published in 2007 as a first author (a) concerning brand attitude indexed in the Scopus database. The respective author has a total of 134 articles published with an overall h-index of 34; however, the current study does not consider due to focusing on brand attitude. The list is followed by Chang, Chingching (15, 11), Muehling, Darrel D. (14, 10). These articles are among the main articles concerning product and brand attitude, and other allied venues and authors primarily refer to it. Based on citations, De Pelsmacker, P. is at the top of the list with 658 citations, followed by Muehling, Darrel D. with 560 citations, Cauberghe, Verolien with 541 citations, and Merrilees, Bill with 493 citations.

**Table 2:** List of most prolific authors in brand attitude.

#	Name of Author	Author ID	1st article year and Author position	Country	Articles Published	h-index	Total Citations	Academic Institution
1	De Pelsmacker, P.	6602498154	2007a	BG	19	10	658	University of Antwerp, Faculty of Business and Economics, Marketing Department, Prinsstraat 13, Antwerp
2	Chang, Chingching	7407043428	2004a	TN	15	11	383	Academia Sinica, Research Center for Humanities and Social Sciences, Taipei
3	Muehling, Darrel D.	6602549875	1987a	UN	14	10	560	Washington State University, College of Business, Pullman
4	Dens, Nathalie	23988285100	2010a	BG	12	7	212	University of Antwerp, Faculty of Business and Economics, Marketing Department, Prinsstraat 13, Antwerp
5	Merrilees, Bill	57213527598	2002a	AUS	10	10	493	Griffith Business School, Brisbane
6	Cauberghe, Verolien	23567464000	2008a	BG	9	7	541	Department of Communication Sciences, Ghent University, Gent
7	Smit, Edith G.	14069091300	2015e	NL	9	7	142	Amsterdam School of Communication Research (ASCoR), University of Amsterdam, Amsterdam
8	van Reijmersdal, Eva Adriana	16308348100	2012b	NL	9	7	401	Amsterdam School of Communication Research (ASCoR), University of Amsterdam, Amsterdam
9	Laczniak, Russell N.	6603355324	1988b	US	8	6	234	Iowa State University, Ames
10	Bellman, S.	56228692400	2009a	AUS	7	4	286	UniSA Business, Adelaide

*Belgium (BG), Taiwan (TN), United States (US), Australia (AUS), Netherlands (NL).

### 4.4 Illustrating most productive countries, academic institutions, and collaboration

**Figure 3:**
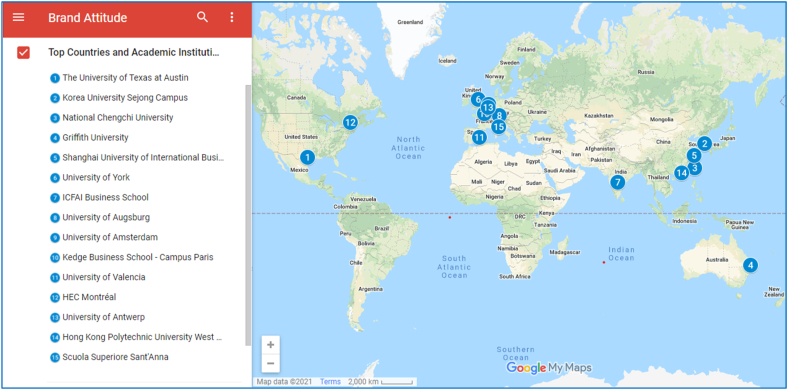
Illustrating top 15 most productive countries and academic institutions considering the brand attitude.

In this sub-section, to provide a holistic view of researchers and their affiliated academic institutions' global geographic spread, the authors had drawn on google map data 2021 indicating their location and placement. Fig. 3 shows the global mapping of the top 15 most productive countries and their academic institutions, along with the names displayed according to their ranking pertaining to brand attitude publications indexed in the Scopus database. The countries and their top academic institutions are further illustrated in Table 3. The google map data shows that the European region is dominant in publishing concerning brand attitude; however, the prolific authors and their academic institutions are mainly clustered in two regions, i.e., Europe and Pacific countries. The other regions include North America and South Asia.

**Table 3:** Top 15 most productive Territories with Educational Institutions in Brand Attitude.

#	Territory	Total articles published by a country TAP	No. of articles of a single country	Single country articles published SAP%	Educational Institution	Total articles published by a given educational institution TAPEI
1	United States	627	430	68.581	The University of Texas at Austin	20
2	South Korea	148	89	60.135	Sejong University	14
3	Taiwan	113	85	75.221	National Chengchi University	20
4	Australia	96	46	47.917	Griffith University	21
5	China	85	29	34.118	Shanghai University	6
6	United Kingdom	76	18	23.684	University of York	5
7	India	67	57	85.075	IBS Hyderabad	10
8	Germany	58	28	48.276	Universität Augsburg	5
9	Netherlands	57	43	75.439	Universiteit van Amsterdam	32
10	France	54	19	35.185	KEDGE Business School	8
11	Spain	47	23	48.936	Universitat de València	8
12	Canada	45	16	35.556	HEC Montréal	6
13	Belgium	40	26	65.000	Universiteit Antwerpen	22
14	Hong Kong	30	8	26.667	Hong Kong Polytechnic University	7
15	Italy	30	15	50.000	Sant'Anna Scuola Universitaria Superiore Pisa	3

Table 3 portrays the most productive countries along with academic institutions publishing and contributing to the field of brand attitude. This study focuses only on reporting academic institutions for the purpose of brevity and being an academician and therefore ignores the non-academic institutions. The United States, with 627 documents, is at the top of the list leading with a high margin considering entire documents published focusing on brand attitude. The list is followed by South Korea (148), Taiwan (113), Australia (96). The list depicts that the researchers from developed economies have a dominant share in and among the top 15 economies, while researchers from only two emerging economies, such as China (85) and India (67), could make a space in the list. Since the developed economies are highly industrialized and developed, they spent much more on their research and development concerning their product brands and the attitude of the customers and consumers towards the brands. Furthermore, due to global trade openness, there is stiff competition existing in capturing market share. Therefore, it is needed to have updated knowledge of consumer brand attitudes to place their product further. In addition, the emerging economies following footprints of developed economies are also investing towards research and development and concerning towards brand attitude to sustain competition in the global marketplace.

Moreover, the column of single country documents published, and its percentage show the country's research productivity alone by excluding all the articles published with other countries. Based on this, India leads the list by producing 57 articles with 85.075%, followed by the Netherlands (43, 75.439%) and Taiwan (85, 75.221%). This depicts that these economies rely more on collaboration among country authors with respect to across economies. However, the United States, with 430 articles, followed by South Korea (89) and Taiwan (85), produces more than India and others; however, the percentage is less due to a high number of documents produced. Table 3 also shows the metric of top academic institutions with the Universiteit van Amsterdam, the Netherlands, in a lead role with 32 documents published, followed by Universiteit Antwerpen, Belgium (22), Griffith University, Australia (21). Similarly, The University of Texas at Austin, United States, and National Chengchi University, Taiwan produced (20) documents each.

It is suggested that academic institutions should focus on cross-border collaboration to extend the network of authors and countries, bring more quality to the articles produced, share expertise, timely and valid data collection. Moreover, since the majority of the branded products are also sent to the markets of developing or emerging economies, therefore for developed countries, researchers to collect handful and reliable data external collaboration is highly recommended. Furthermore, such collaboration helps in an increased number of documents and citations, ranking high for the authors and affiliated academic institutions. Moreover, for instance, India is a big consumer market with its high population figures but producing intra country publications needs to adopt cross-border collaboration to extend the knowledge regarding brand attitude. Finally, the authors found that two universities listed in Table 3 are placed in the world's top 100 university rankings, i.e., Hong Kong Polytechnic University (66) and The University of Texas at Austin (67) in Ref. [52]. This shows that the brand attitude is in vogue and has consideration at top academic institutions worldwide.

**Figure 4:**
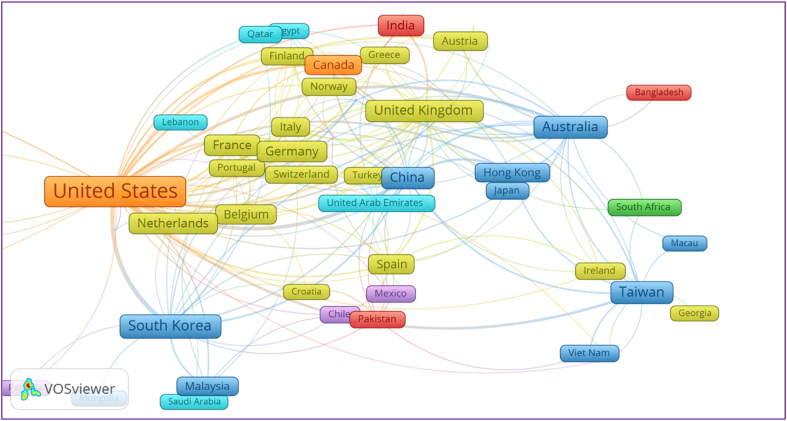
Bibliometric knowledge map built on co-authorship with network visualization mode. This can be accessed using Online URL https://bit.ly/3iAp6VO.

Fig. 4 reveals the countries spread on co-author analysis and are clustered into seven regions by adopting to distribution by Refs. [40,53]. This closeness of the countries shows their connectedness, and the thickness of the line shows the link strength between the two countries, i.e., the thin line pertains to a weaker relationship and vice versa. Cluster 4 consisting of European and Central Asian countries share the significant portion in publications with 27 countries considering brand attitude, followed by East Asia and The Pacific (14), Middle East and North Africa (09), Latin America and The Caribbean (08), Africa (03), South Asia (03), and North America (2) countries. Our results of co-authorship analysis described that the United States has highest linkages than any other country with 47 links (linked to 47 other countries), 231 total link strength (231 times co-authorship) utilizing 624 documents. The pictorial size of the frames also gives a birds-eye view of the countries. This is followed by the United Kingdom (26 links, 73 total link strength), Australia (22 links, 61 total link strength), South Korea (18 links, 72 total link strength), China (18 links, 69 total link strength), and Taiwan (13 links, 34 total link strength), etc. Further details can be explored by the online link provided in Fig. 4 caption. The number of international links and link strength depends on various determinants such as the phenomenon of interest, the industry needs, nature of the problem, research and development funds, research students' and international visiting researchers’ mobility across borders, and research partners' diversity name a few.

### 4.5 Author keywords, hotspots, and future conduits

**Figure 5:**
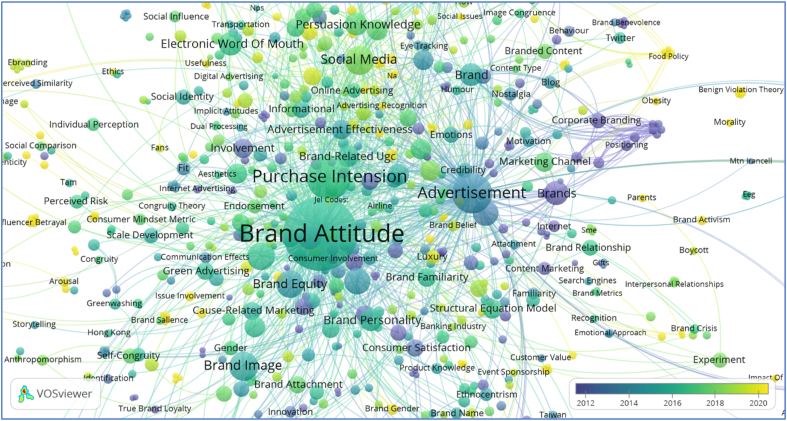
Knowledge map-overlay visualization mode of brand attitude based on author keywords co-occurrence online accessible through https://bit.ly/2Uerus0.

**Figure 6:**
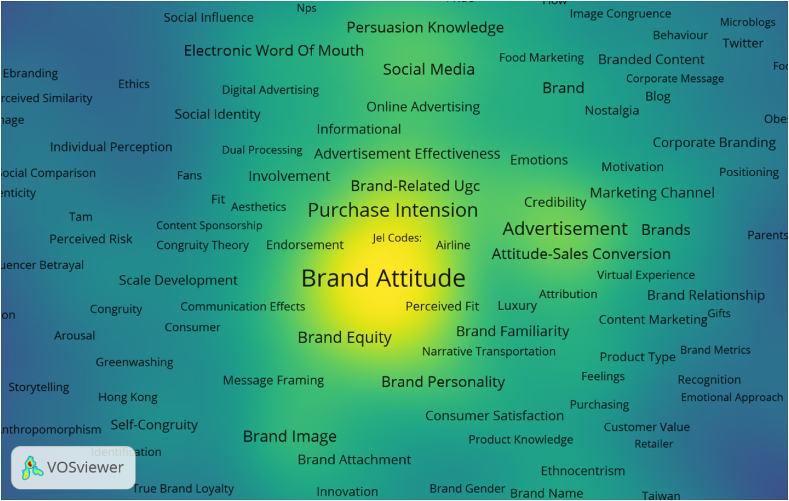
Bibliometric knowledge map-density visualization mode of brand attitude based on keywords co-occurrence online accessible through https://bit.ly/2Uerus0.

The Scopus file of 1497 articles is exported into the VOSviewer to conduct co-occurrence analysis and to suggest future hotspot areas for the researchers considering brand attitude, as shown in Figs. 5 and 6. A total of 3380 keywords were recorded. The VOSviewer has a threshold value of considering 1000 keywords at a time, and it selects the most occurring keywords by default. After re-labeling synonymic words and phrases, the final keywords shown are 979 in number, meeting the minimum limit of a single occurrence. The results of the VOSviewer, as shown in Fig. 5, depict that the brand attitude is the most prominent keyword with 606 links, 1331 total link strength, and 453 occurrences. The other most allied keywords are purchase intention (235 links, 436 total link strength, and 126 occurrences), advertisement (234 links, 375 total link strength, and 101 occurrences), brand image (92 links, 154 total link strength, and 44 occurrences), attitude (90 links, 117 total link strength, and 39 occurrences), social media (89 links, 134 total link strength, and 44 occurrences), brand equity (74 links, 119 total link strength, and 39 occurrences), purchase behavior (71 links, 98 total link strength, and 36 occurrences) etc.

The bibliometric knowledge maps in Figs. 5 and 6 shows that brand attitude is a multidimensional concept linked many such as purchase intention (235 links, 436 link strength, 126 occurrences), advertisement (234 links, 375 link strength, 101 occurrences), brand image (92 links, 154 link strength, 44 occurrences), social media (89 links, 134 link strength, 44 occurrences) brand equity (74 links, 119 link strength, 39 occurrences), purchase behavior (71 links, 98 link strength, 36 occurrences), brand loyalty (45 links, 69 link strength, 26 occurrences). The giant bubbles of brand attitude, purchase intention, and advertisement shows that these are the most linked and researched areas considering brand attitude. It is evident from the fact that, customers are motivated by social media advertisement and due to positive reviews and trustworthy celebrity endorsements [54]. Similarly, brand attitude is also linked to other hotspots such as brand identification through brand image, brand personality, customer attitude, brand preference, and brand management.

Fig. 6 shows the density mode of the keywords analyzing brand attitude and co-occurrence of other related keywords. This knowledge map shows dep density around keywords such as brand attitude and extends towards brand equity, purchase intention etc. The other small density is visible around keywords such as advertisement, social media, persuasion knowledge, and brand image.

The knowledge maps obtained from bibliometric analysis portray emerging trends in any research area [33]. The function of the average year of publications of VOSviewer signifies the hotspots, while the smaller number of occurrences specifies the niche area [21,39]. Developing on this background, Table 4 illustrates the potential emerging hotspots pertaining to brand attitude. The results displayed in Table 4 shows that the most potential terms are, for instance, brand avoidance with the average publication year of 2021, number of occurrences one time, and both average and normalized citation equals none. The second important concept is subtle, with the average publication year of 2021 and the number of occurrences. The concept of Subtle is an attempt to make the consumers aware of the brand without their knowledge because the stimuli are embedded in the context (Chen, 2018). Similarly, this is followed by media priming (2021, 1), which is the introduction of a stimulus and the effects generated by that stimulus, which might include future changes in beliefs, attitudes, and behaviors, are referred to as priming [55].

**Table 4:** Emerging hotspots considering brand attitude.

Keyword	Total Link Strength	No. of Occurrences	Avg. Publication year	Avg. Citations	Normalized Citations
Based on Average Publication Year					
Brand Avoidance	7	1	2021	0.00	0.00
Subtle	5	1	2021	0.00	0.00
Media Priming	5	1	2021	0.00	0.00
Co-Branding	7	1	2021	3.00	3.58
Brand Betrayal	10	2	2021	3.50	4.17
Influencer Marketing	8	1	2021	0.00	0.00
Consumer Ethnocentrism	5	2	2021	1.00	1.19
Product-Harm Crisis	8	2	2021	0.00	0.00
Environmental Sustainability	8	1	2021	9.00	10.73
Environmental Policy	7	1	2021	0.00	0.00
Brand Activism	4	3	2020.67	2.00	0.63
Sensory Marketing	1	2	2020.50	6.00	3.20
Fan Identification	7	2	2020.50	2.50	0.78
Brand Authenticity	9	4	2020.50	2.75	1.13
Brand Identification	12	3	2020	7.00	1.07
Brand Choice	17	3	2020	2.67	0.83
Consumer Brand Engagement	2	2	2020	2.50	0.78
Sns Marketing	7	3	2020	0.33	0.10
Brand Engagement	6	3	2019.67	5.00	2.37
Package Design	4	3	2019.67	7.67	0.41
Brand Competence	8	2	2019.50	1.00	0.21
Brand Communities	12	2	2019.50	3.50	0.35
Explicit and Implicit Brand Attitude	3	1	2018	1.00	0.10

Moreover, the list continues with the hotpot keyword Co-Branding (2021, 1), which is when two or more brands or companies form a relationship to launch their co-brand, co-branding is developed [56]. The next emerging hotspot for future research avenues considering brand attitude is Brand Betrayal (2021, 2), a state that occurs when a brand with which one has previously formed a strong self–brand connection breaks a relationship by committing a moral transgression [57]. Likewise, the keywords Influencer Marketing (2021, 1) portray the strategy of utilizing social media influencers to promote and engage with a brand's message. Social media influencers have accumulated a significant social network of people who follow them [54,58]. Finally, Table 4 show the keywords implicit and explicit brand attitude occurring at cluster number 25, with total link strength 3 and number of occurrences 1. Since, the smaller number of occurrences specifies the niche area [39], therefore, it needs serious attention of the researchers. A part of the studies that investigated the respondents' attitudes, they also classify attitudes into implicit attitudes and explicit attitudes. Explicit attitude is the attitudes that are at the conscious level. It is a construct that social psychologists often attempt to test using questionnaires or interviews [59]. Explicit attitudes are also called deliberative, conscious, or intentional [60]. On the other hand, implicit attitude is the unconscious attitudes, which may be described as evaluative responses to an item that is not always susceptible to self-reflection [59]. Associative, unconscious, or automatic attitudes are all terms used to describe implicit attitudes [60]. In the case of consumers' brand attitudes, there is a scarcity of studies that investigated the implicit consumers' brand attitudes. Also, there are very few studies that look at the consumers' implicit and explicit brand attitudes e.g. Ref. [58].

Similarly, while implicit attitudes cannot replace conventional explicit attitudes, they do provide communication researchers with valuable information on affect, motivation, and emotion [61]. In some cases, implicit attitudes are better predictors of actual behaviors than explicit attitudes [60]. It has been discovered that in forecasting probable voting behavior, implicit attitudes offer better predictions than explicit ones. Since implicit measures have been shown to more accurately assess those automatic behaviors, which result from implicit attitudes, than explicit attitudes. Thus, when analyzing consumers' behavior, it is important to consider both implicit and explicit brand attitudes [62]. Equally, Table 4 illustrates all the hotspot emerging new avenues for future research linking it to brand attitude could enrich the literature. The keywords such as brand activism, environmental sustainability, sensory marketing, brand identification, brand choice, package design, implicit and explicit brand attitude, and brand competence are the other emerging avenues and future conduits attracting both the academicians, researchers and practitioners, and policymakers in order to obtain long term environment and growth sustainability.

## 5 Conclusion

The current study signifies the historical trend and developments considering brand attitude based on 1497 publications extracted from the Scopus database for the time period 1944 to August 2021 for more than 76 years. The first publication indexed in the Scopus database is in the year 1944, following which there is very slow growth in the number of publications concerning brand attitude until 2008, then mild increase which further improved after 2012 till recent. The spike in cumulative publications can be seen after 2012 in Fig. 2. The researchers from the United States lead the study area with bulk publications than any other country, followed by South Korea, Taiwan, and Australia. However, globally the developed economies are extensively focusing on brand attitude compared to developing or emerging economies. The placement of China and India in the list signifies the importance of research about the brand attitude in emerging economies. Such focus can open venues and multiply publications many folds through cross-border collaborations. This may help in enriching the literature from emerging and developing world perspectives towards brand attitude. Based on the current trend and emerging hotspots, this study suggests the future avenues of research considering brand attitude as brand avoidance, subtle, media priming, co-branding, brand activism, brand identification, brand choice, environmental sustainability, brand choice, and brand competence etc. Various scholars, researchers, organization research and development centers, and government bodies shall focus these avenues for policy recommendations in securing the environment and sustainable development goals both in the long-run and short-run. In the case of consumers' brand attitudes, there is a scarcity of studies that investigated the implicit consumers' brand attitudes. Also, there are very few studies that look at the consumers' implicit and explicit brand attitudes. More specifically, future studies can focus on this area concerning brand attitude.

Moreover, this bibliometric analysis identified overarching patterns and discursive challenges in Brand attitude literature compared to prior literature with additional insights. The study indicated the importance of brand attitude and its outcomes covering all the relevant areas of diversity research and pointed out that Brand attitude is one of the most valuable assets for a company to achieve competitive advantages and increase customer purchase intention for business profitability.

This study has contributed several theoretical implications. First, to our best knowledge, this is the first study that has explored the root of brand attitude literature by reviewing the previous literature from 1944 to 2021 using bibliometric analysis. Second, this study has analyzed the most prolific articles containing the most productive authors and their affiliations, keywords, and influential journals, along with future research in the brand attitude research that has significantly addressed the prominent work, theoretical insights and guided the directions for future researchers. This study has integrated existing studies from the all the previous literature available on brand attitude and provided comprehensive knowledge, current research trends on brand attitude literature to suggest future research directions. Based on this study's findings, future researchers can easily identify the most influential published articles, journals, prominent authors and their seminal work in this area for identifying research gaps and new insights. To conclude, this study has identified gaps and made novel contributions to the growing body of literature. From the managerial perspective, this study has provided several practical implications and valuable insights. This study can help managers in making decisions about investing on aspects of brand attitude building to develop loyal and dedicated customers, which will in turn result in competitive advantage and business success. Investigating the factors that influence brand attitude in an empirical study could also be a useful contribution to managerial practice.

### 5.1 Limitation of the study

Despite tremendous efforts and efficient use of software, there persist chance of some limitations in each study, which is also possible in ours. One possible limitation could be the journal space for articles that limit us to discuss the main features of the bibliometric analysis rather than going in-depth and discussing many things. Secondly, according to the authors' best knowledge, this is the first study, laying the foundation for other studies; other researchers can use a combination of other keywords with ours and focus on more perspectives than the one discussed here. Finally, we have focused on the Scopus database, and other studies can focus on the Web of Science database or both Scopus and WOS to give more coverage to the articles.

## Data availability statement

The data can be retrieved using the key provided in the body of the text, while for bibliometric knowledge maps, we have provided online links to reach out to the data.

## Declaration of competing interest

The authors of this manuscript declare that they have no conflict of interest concerning its drafting, publication, or application.

## Funding

The authors received no direct funding for this research.
